# MiR-223 and MiR-186 Are Associated with Long-Term Mortality after Myocardial Infarction

**DOI:** 10.3390/biom12091243

**Published:** 2022-09-06

**Authors:** Meyer Elbaz, Julien Faccini, Clémence Laperche, Marie-Hélène Grazide, Jean-Bernard Ruidavets, Cécile Vindis

**Affiliations:** 1 Department of Cardiology, Rangueil University Hospital, 31400 Toulouse, France; 2 Center for Clinical Investigation (CIC1436)/CARDIOMET, Rangueil University Hospital, 31400 Toulouse, France; 3 INSERM UMR 1048, 31400 Toulouse, France; 4 Department of Epidemiology, INSERM UMR 1027, 31400 Toulouse, France

**Keywords:** microRNA, biomarker, myocardial infarction, cardiovascular disease

## Abstract

Background—The identification and stratification of patients at risk of fatal outcomes after myocardial infarction (MI) is of considerable interest to guide secondary prevention therapies. Currently, no accurate biomarkers are available to identify subjects who are at risk of suffering acute manifestations of coronary heart disease as well as to predict adverse events after MI. Non-coding circulating microRNAs (miRNAs) have been proposed as novel diagnostic and prognostic biomarkers in cardiovascular diseases. The aims of the study were to investigate the clinical value of a panel of circulating miRNAs as accurate biomarkers associated with MI and mortality risk prediction in patients with documented MI. Methods and Results—seven circulating plasma miRNAs were analyzed in 67 MI patients and 80 control subjects at a high cardiovascular risk but without known coronary diseases. Multivariate logistic regression analyses demonstrated that six miRNAs were independently associated with MI occurrence. Among them, miR-223 and miR-186 reliably predicted long-term mortality in MI patients, in particular miR-223 (HR 1.57 per one-unit increase, *p* = 0.02), after left ventricular ejection fraction (LVEF) adjustment. Kaplan–Meier survival analyses provided a predictive threshold value of miR-223 expression (*p* = 0.028) for long-term mortality. Conclusions—Circulating miR-223 and miR-186 are promising predictive biomarkers for long-term mortality after MI.

## 1. Introduction

Coronary heart disease (CHD) ranks as the most prevalent cause of death in both men and women. Despite improvements in acute care and secondary prevention after myocardial infarction (MI), the burden of CHD is expected to continue due to increased obesity, diabetes, inactivity and also to population aging [1]. Data indicate that one-third of patients die before coming to the hospital, at least 5–10% of survivor patients die within the first 12 months after their MI, and close to 30% of patients need readmission within the same year [2,3]. Currently, no high-quality soluble biomarkers are available to accurately identify subjects who are at risk of suffering acute manifestations of CHD as well as to predict fatal clinical outcome after MI. The dysregulation of specific microRNAs (miRNAs) has been proposed to be a diagnostic biomarker of acute MI [4] and to influence post-MI complications during follow-up [5].

MiRNAs are a class of small endogenous noncoding single-stranded RNA (19–24 nucleotides), which are essential post-transcriptional modulators of gene expression by binding to the 3’ untranslated region of specific target genes, thereby leading to suppression or translational repression [6]. It is now established that miRNAs are important regulators and fine-tuners of both physiological and pathological cellular processes, including those relevant to the cardiovascular system [7,8]. MiRNAs are expressed intracellularly and could be secreted in body fluids (blood circulation, urine or saliva), and are usually associated with exosomes, apoptotic bodies, lipoproteins or protein complexes. Circulating miRNAs remain stable since they are protected from plasma ribonucleases by their carriers, including lipid vesicles or protein conjugates, such as Argonaute 2 or other ribonucleoproteins [9]. Indeed, it has been demonstrated that miRNAs are highly stable in boiling water, prolonged room temperature incubation, or repeated freeze–thawing [10]. 

Regardless of their function, the interest of miRNAs for biomarkers in acute or chronic cardiovascular diseases has been growing over the past few years [11]. Recently, we have identified three circulating plasma miRNAs associated with the diagnosis of coronary artery disease (CAD) [12] as well as a powerful miRNA-based signature improving the risk prediction of acute coronary syndrome (ACS) [13]. Based on the results of our previous study [13], we sought to validate the accuracy of the miRNA signature associated with the risk of MI in a population of patients who experienced MI, compared to a population of subjects without known *CAD*, but with *high* cardiovascular *risk*. We also investigated whether these selected miRNAs could also be predictors of long-term mortality after MI. Among the tested circulating miRNAs, our present findings highlight miR-223 and miR-186 as promising biomarkers associated with the risk of MI and long-term mortality after MI.

## 2. Materials and Methods

### 2.1. Study Population

The case–control study comprised 173 subjects admitted to the department of Interventional Cardiology and Prevention of Cardiovascular Disease (Toulouse University Hospital Center, Toulouse, France). Cases (70 men and women aged >18 years) were patients with diagnosis of acute MI as previously defined [14], admitted within 48 h of symptom onset and consecutively recruited between November and December 2015 (DC-2008-4623). Blood samples were collected within 2~3 days after the acute ischemic event. The control population consisted of 103 subjects (men and women) from a previously described case–control study (NCT02405468, [13]). Control subjects were at a high cardiovascular risk of primary prevention as defined by the presence of at least two of the following risk factors: treated dyslipidemia, treated hypertension, treated diabetes mellitus, or current smoking. Control subjects were free of known coronary disease on the basis of clinical data, ECG, echocardiogram, and a cardiac stress test. Exclusion criteria for both groups included the following: infectious disease within 1 week before enrollment, immunocompromised patients, antibiotic treatment within 1 month before inclusion, chronic viral infection, chronic inflammatory bowel disease, renal failure (estimated glomerular filtration rate < 50 mL/min per 1.73 m^2^), and pregnancy. The full demographic, clinical and biological data were available from 67 cases and 80 control subjects, with a 6 year-follow up for mortality. Written informed consent for the possibility that their anonymized clinical data could be used for scientific purposes was obtained from each patient. Long-term follow-up was obtained with the interrogation of municipal registry offices, physicians and by direct contact with the patients or their families. The study complies with the Declaration of Helsinki. The protocol was reviewed and approved by the regional board of health authorities (Agence Régionale de Santé-Midi-Pyrénées) and the local Ethics Committee.

### 2.2. Total RNA Isolation and Quality Control

Total RNA was extracted from 200 µL of EDTA–plasma using the miRNeasy serum/plasma kit (Qiagen SAS, Courtaboeuf, France), according to the manufacturer’s instructions. Briefly, 5 µL of 1 nM synthetic *Caenorhabditis elegans* miR-39-3p (cel-miR-39-3p) miRVana miRNA Mimic (MC10956, Fisher Scientific, Illkirch, France) and *Caenorhabditis elegans* miR-39-5p (cel-miR-39-5p) miRVana miRNA Mimic (MC20682, Fisher Scientific, Illkirch, France) was spiked into each plasma sample to monitor the efficiency of miRNA recovery and to normalize miRNA expression in the subsequent real time PCR as described previously [12]. The quantity and quality of RNA were assessed with a NanoDrop spectrophotometer (NanoDrop Technologies, Wilmington, DE, USA).

### 2.3. MiRNA Expression Analysis

The expression of the selected plasma miRNAs was quantified with RT-qPCR. RT and pre-amplification were performed on all samples using a custom RT primer pool and custom preamp primer pool (PN 4427975, Fisher Scientific SAS, Illkirch, France) with the recommended protocol (LTC publication PN 4465407 Rev. B). The cDNA was prepared with 3 µL of total RNA per sample using the TaqMan MicroRNA Reverse Transcription Kit (PN 4366596, Fisher Scientific SAS, Illkirch, France) and custom primer pool in 15 μL final volume. The RT reaction was thermal-cycled (30 min at 16 °C, 30 min at 42 °C) and the enzyme was inactivated at 85 °C for 5 min. Pre-amplification was performed using 2.5 μL of the RT reaction as previously described [13]. The pre-amplification product was diluted 1:8 in TE then diluted 1:100 in 1× TaqMan Universal Master Mix II (PN 4440040, Fisher Scientific SAS, Illkirch, France) before loading on 96-well plate. The 96-well plates were sealed, spun and then run on a StepOne Plus Real Time PCR system using universal cycling conditions (10 min at 95 °C; 15 s at 95 °C, 1 min at 60 °C, 40 cycles). Ct values were normalized to cel-miR-39-3p and cel-miR-39-5p and the relative expression of the miRNAs was calculated by the 2^−ΔΔCt^, ^−[Ct(miRNA target) – (meanCt(miRNA control))]^ method as described [15]. If the Ct values differed by more than six Ct between an individual compared to the average of the others, a new extraction was performed. Data were analyzed using the Applied Biosystems analysis Software version 3.1 (Fisher Scientific SAS, Illkirch, France) on the Thermo Fisher Scientific cloud.

### 2.4. Statistical Analysis

Statistical analyses were carried out using the statistical SAS software package (SAS 9.4 Institute, Cary, NC, USA). All tests were two-tailed and were considered significant at the 0.05 level. Continuous variables were summarized as means and standard deviations. Categorical variables are presented as proportions. In a univariate analysis, categorical variables were compared with the Chi-squared test (or Fischer’s exact test when necessary). The comparison between the mean values of quantitative variables was performed by using the Student’s *t*-test. We used the Shapiro–Wilk’s and Levene’s tests to test the normality of distribution of residuals and the homogeneity of variances, respectively. When the basic assumptions of the Student’s *t*-test were not satisfied, we performed a logarithmic transformation of the variables or a Wilcoxon–Mann–Whitney test. The expression levels of miRNA in controls and patients are shown in descriptive statistics as box plots. Logistic regression analyses were carried out using miRNA variables categorized into tertiles. A penalized likelihood-based method (Firth correction) has been applied to adjust for the small sample size bias. Unadjusted and multivariate adjusted models were performed with systematic adjustment for gender, age, obesity, dyslipidemia, hypertension and smoking. The SAS^®^ Statistical software version 14.2 (SAS Institute Inc., Cary, NC, USA) was used to assess the ROC curves and to compare areas under the ROC curves, using the calculations from [16]. The contribution of each selected circulating miRNA was tested against the basic clinical model. The cumulative survival of patients was determined by the Kaplan–Meier method and compared using the Log-rank test. Univariate and multivariate Cox regression models were used to investigate the association between miRNA variables and mortality during the follow-up and to determine hazard ratios (HR) and 95% confidence intervals (95% CI). Regression analyses were performed with polynomial models (quadratic and cubic) to examine for a possible nonlinear relation between continuous miRNA variables and mortality. When each miRNA was significantly associated with mortality in bivariate analysis a further adjustment on left ventricular ejection fraction (LVEF) was performed. The proportional-hazard assumption was tested by the “log-log” method, plotting (-ln[-ln(survival)]) for each category of a nominal covariate, versus ln(analysis time). None of the assumptions could be rejected.

## 3. Results

### 3.1. Population Characteristics and MiRNA Profile Analysis

**Figure 1:**
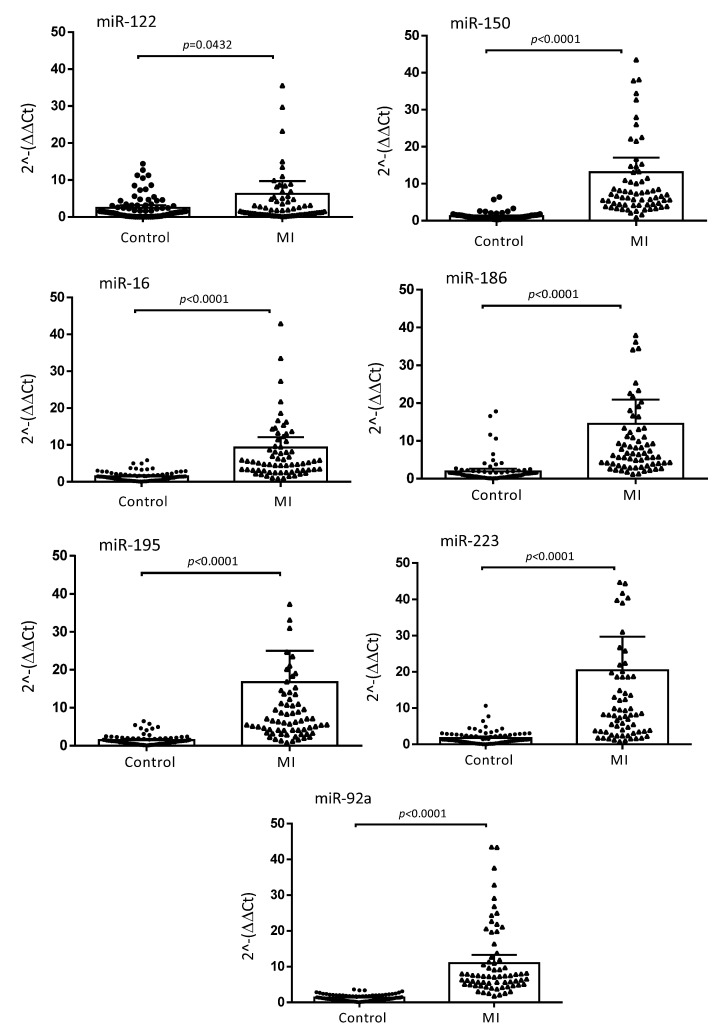
Relative expression of plasma miRNA levels in MI and control populations. The ΔΔCt method was used to quantify the relative miRNA expression of miR-122, miR-150, miR-16, miR-186, miR-195, miR-223 and miR-92a. Ct values were normalized to cel-miR-39-3p and cel-miR-39-5p and the relative expression of the miRNAs was calculated by the 2^−ΔΔCt^, ^−[Ct(miRNA target) − (meanCt(miRNA control))]^ method. Data are presented as means with 95% CI (CI; confidence interval). *p* values were calculated with the Wilcoxon–Mann–Whitney test.

The baseline characteristics of the MI and control subjects are presented in Table 1. MI patients were older (*p* < 0.01) and more were smokers (*p* < 0.001) as compared to the control subjects. The high prevalence of dyslipidemia, hypertension or obesity, as well as the significant differences in the medical treatments found in the control group, were explained by their recruitment in the department of Prevention of Cardiovascular Disease (Toulouse University Hospital Center, Toulouse, France). Based on a miRNA profile identified in our previous work [13], the following seven miRNA candidates were quantified by qPCR in plasma samples of the studied subjects: miR-122, miR-150, miR-16, miR-186, miR-195, miR-223 and miR-92a. The miRNAs were successfully validated and, in agreement with our previous results, we confirmed that the expression levels of the seven miRNAs were significantly higher in MI patients when compared to control subjects. The calculated fold changes were 6.26 (*p* = 0.0432) for miR-122; 13.1 (*p* < 0.0001) for miR-150; 9.3 (*p* < 0.0001) for miR-16; 14.5 (*p* < 0.0001) for miR-186; 16.77 (*p* < 0.0001) for miR-195; 20.47 (*p* < 0.0001) for miR-223; 10.97 (*p* < 0.0001) for miR-92a (Figure 1).

### 3.2. Association of MiRNA Candidates with the Risk of MI

The results presented in Table 2 show the crude estimates and adjusted associations of the seven miRNAs with the risk of MI. The univariate analysis revealed that miR-150, miR-16, miR-186, miR-195, miR-223 and miR-92a were significantly associated with the risk of MI, while no significant association was found for miR-122. After adjustment for traditional risk factors, including gender, age, obesity, dyslipidemia, hypertension and smoking, the associations remained statistically significant. For miR-150 (OR = 6.67, *p* = 0.01), miR-16 (OR = 5.27, *p* = 0.001), miR-186 (OR = 4.22, *p* = 0.001), miR-195 (OR = 5.9, *p* = 0.001), miR-223 (OR = 4.27, *p* = 0.001) and miR-92a (OR = 8.86, *p* = 0.001), the probability of having MI was between four and eight times higher in the third tertile in comparison with the first one. We then assessed whether miR-150, miR-16, miR-195, miR-186, miR-223 and miR-92a could increase the predictive performance of the basic clinical model. A receiver operating characteristic (ROC) curve analysis was performed with the six miRNA candidates. Data in Table 3 show the individual performance (area under the curve, AUC) of each miRNA and the improvement of the clinical model predictive performance. Addition of each miRNA to the clinical model led to a significant raise in the AUC from 0.914 to 0.996 for miR-150, to 0.981 for miR-16, to 0.973 for miR-186, to 0.986 for miR-195, to 0.968 for miR-223 and to 0.999 for miR-92a.

### 3.3. MiR-223 and MiR-186 Are Associated with Long-Term Mortality after MI

**Figure 2:**
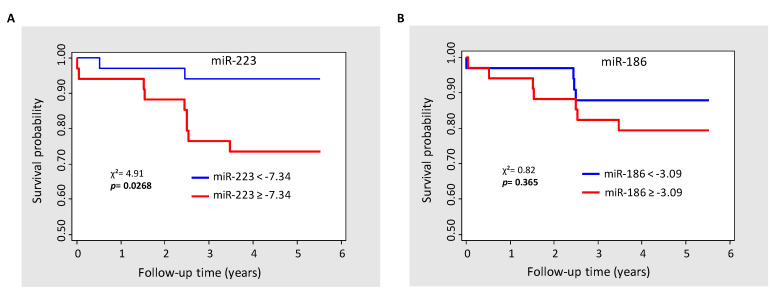
Kaplan–Meier survival estimate according to the median of miR-223 (**A**) and miR-186 (**B**). χ^2^, Chi-squared test.

During the 6-year follow-up period, 11 deaths (16.4%) occurred in the 67 studied MI patients and two deaths (2.5%) in the 80 controls (the number of events in the control group was too small to perform statistical analysis). The relative expression levels of the six selected miRNAs in the “alive group” (MI patients living at the end of the follow-up) and the “deceased group” (MI patients who died during the follow-up) are presented in Table 4. Among the six tested miRNAs, miR-223 and miR-186 had significantly increased expression levels in the “deceased group” compared to the “alive group”. Notably, Cox regression analyses showed that the levels of miR-223 (HR 1.75 per one-unit increase, *p* = 0.0045) and miR-186 (HR 1.56 per one-unit increase, *p* = 0.025) were significantly correlated with all causes of mortality after MI (Table 5). Remarkably, after adjustment for LVEF, which is one of the major mortality predictors following MI, the prognostic value of miR-223 level remained statistically significant (HR 1.57 per one-unit increase, *p* = 0.02) and close to significativity for miR-186 (HR 1.41 per one-unit increase, *p* = 0.065). The analysis of Kaplan–Meier survival curves according to the median threshold value of miR-223 (Figure 2A) confirmed the high predictive value of miR-223 (*p* = 0.0268) for long-term mortality in MI patients, but not for miR-186 (*p* = 0.365) (Figure 2B).

The ΔΔCt method was used to quantify relative miRNA expression in MI patients who died during the follow-up and MI patients living at the end of the follow-up. Ct values were normalized to cel-miR-39-3p and cel-miR-39-5p and the relative expression of the miRNAs was calculated by the 2^−ΔΔCt^, ^−[Ct(miRNA target) − (meanCt(miRNA control))]^ method. Data are presented as mean ± SEM. *p* values were calculated with the Wilcoxon–Mann–Whitney test.

## 4. Discussion

The discovery of reliable and sensitive biomarkers in CHD remains a critical clinical need. Circulating non-coding RNAs, especially the miRNA family, are currently being explored as complementary biomarkers of cardiac injury. Indeed, our results support numerous studies reporting deregulated levels of circulating miRNAs associated with cardiovascular diseases, including MI or heart failure [11,17]. In a previous study comparing a population of ACS patients and high-risk control subjects, we showed that a panel of circulating miRNAs was differentially expressed and independently associated with the risk of ACS [13]. Our present work further confirms our previous results since we validated their differential expression in another population of MI patients. Furthermore, out of six tested miRNAs, we showed the association of six of them (miR-150, miR-16, miR-186, miR-195, miR-223 and miR-92a) with the risk of MI in high-risk populations with clinically unconfirmed coronary disease. The incremental value of the six miRNA candidates was also highlighted by a significantly increased AUC when miRNAs were added to a clinical prediction model. Thus, our data underline the clinical significance of miR-150, miR-16, miR-186, miR-195, miR-223 and miR-92a as promising complementary biomarkers associated with the risk of MI. 

Moreover, our data highlight the role of miR-223 and miR-186 in predicting mortality, since both miRNAs were significantly highly expressed in the deceased MI patients when compared to the survivors. Additionally, Cox regression analyses indicated a strong association between miR-223 and miR-186 levels and mortality after MI, independently of LVEF for miR-223. Based on Kaplan–Meier survival analyses, we also reported a predictive threshold value for miR-223. Thus, plasma miR-223 may be considered as an independent predictive factor for long-term mortality after MI. 

Vascular and myocardial miRNAs could be selectively released into the circulation in response to cardiac stress, such as MI. Whether the increase in circulating miR-223 and miR-186 in MI patients is a consequence of coronary vasculature or heart-enriched miRNA release is presently unknown. Our findings suggest that mortality risk prediction associated with miR-223 is more likely linked to coronary vasculature dysfunction, since the predictive value is independent of LVEF.

Previous studies have reported that circulating miR-186 could be considered as an emerging diagnostic biomarker for the early phase of acute MI and a prognostic biomarker in ACS patients after percutaneous coronary intervention [18]. Physiologically, miR-186 is considered as an anti-angiogenic miRNA. MiR-186 has been implicated in response to hypoxia via the downregulation of the hypoxia-inducible factor-1α (HIF-1α) a major factor mediating cardio-protection [19]. By targeting HIF-1α, miR-186 restrains vascular endothelial cell proliferation and enhances apoptosis [20], as well as causing glycolysis inhibition by impairing glucose intake and ATP and NADH production [21]. MiR-186 is among the most-expressed miRNAs in the serum of patients with Kawasaki disease (KD), an acute vasculitis that particularly affects the coronary arteries [22]. Its potential role in KD could involve the SMAD pathway, since miR-186 overexpression in HUVECs has been shown to repress SMAD6 expression which then negatively regulates TGF-β signaling, resulting in feedback activation of the MAPK pathway to induce endothelial cell injury [22]. Hence, our data support the hypothesis that miR-186 is associated with fatal outcome after MI and may participate in the progression and the outcomes of MI by affecting hypoxia response and vascular endothelial function.

MiR-223 has been involved in various pathophysiological activities affecting MI by promoting endovascular inflammation and abnormal platelet reactivity [23]. Predominantly found in platelets, but also highly expressed in monocytes, miR-223 is considered to be a myeloid-specific miRNA that operates as a fine tuner of monocyte differentiation and inflammatory responses [24,25]. Since activated platelets correlate with increased mortality in ACS patients, even months and years after the initial event, we can hypothesize that miR-223 levels may be associated with abnormal platelet activation, leading to a higher risk of post-MI mortality. Thus, our findings have reinforced previous works showing an association between increased circulating miR-223 and cardiovascular death risk in symptomatic ACS patients [26]. Pathological cardiac ventricle remodeling contributes to the development of heart failure; interestingly, miR-223 was found to be upregulated in human cardiac tissue from the border zone of the infracted region [27] and in failing human heart samples [28]. Cardiac-specific miR-223 transgenic mice developed cardiac hypertrophy and heart failure whereas miR-223-deficient mice were protected from hypertrophic stimuli, which makes miR-223 an attractive therapeutic target [28]. However, the mechanistic role of miR-223 is controversial; for instance in vitro studies described miR-223 as a pro-apoptotic [29], pro-fibrotic [30] and anti-angiogenic [31] factor, while others have proven that miR-223 protects cardiomyocytes from inflammation [32] and necroptosis [33] induced during ischemia–reperfusion. Moreover, miR-223 regulates cholesterol biosynthesis, uptake, and efflux, thus establishing it as a critical coordinator of cholesterol metabolism which is major risk factor in CAD [34]. HDLs are capable of delivering MiR-223 to recipient cells including hepatic and endothelial cells, mainly through the combination with scavenger receptor class B Type 1 (SR-B1) [35]. Interestingly, plasma HDL-carried miR-223 in ACS patients decreased during the transcoronary passage when compared to patients with stable CAD, suggesting a positive correlation between the uptake of HDL-associated miR-223 by the vascular wall and the severity of coronary disease [36]. However, the function of the uptake of miR-223 by the coronary vasculature in patients with ACS relative to stable CAD subjects is unknown.

Finally, our data highlight the emerging role of miR-223 and miR-186 as being promising biomarkers associated with the risk of MI and with long-term mortality in the secondary prevention settings. Considering that miRNAs are active molecules, our work, if corroborated by other studies, suggests that miR-223 and miR-186 may also represent new targets for therapeutic interventions. Further studies are needed to determine whether the differential expression of circulating miRNAs is a result of modulated intracellular production and/or different release mechanisms. Repeated measures of the plasma miRNAs in MI patients will provide additional information on their stability and activity. Some limitations of our study have to be considered. First, although the sample size had sufficient statistical power to identify significant differences, larger study groups are necessary to confirm the clinical value of miR-223 and miR-186 in long-term mortality after MI but also in high-risk populations. Second, only a selected number of miRNAs based on a preliminary work was evaluated and might only represent a small subset of all differentially regulated miRNAs in patients and controls. Third, it would be informative to measure the baseline expression of the circulating miRNAs before the MI event; however, such information would require a longitudinal study of subjects at a high cardiovascular risk with at least 10-year follow-up.

In conclusion, from a clinical perspective, our study underscores the promising applicability of circulating miR-223 and miR-186 in the cardiovascular risk stratification of CHD patients. The management of MI patients constantly requires novel, more specific and sensitive prognostic and predictive biomarkers. As a prognostic biomarker, miRNAs might help physicians to predict the outcome and overall survival of a MI patient receiving standard or no treatment. As a predictive biomarker, miRNAs could abet the clinician to distinguish which patients are likely or not to benefit from a particular treatment. More generally, the use of miRNAs as non-invasive complementary biomarkers might further help physicians to tailor therapies, improve secondary prevention and lower cardiovascular mortality.

## Figures and Tables

**Table 1:** Baseline characteristics of MI patients and control subjects.

		MI Patients (*n* = 67)	Control Subjects (*n* = 80)	*p* Value
Gender (%)	Male	78	58	0.001
	Female	22	42	
Age (years)		64.9 ± 12.5	60.1 ± 7.9	0.01
Obesity (%)		16.4	32	0.006
Dyslipideamia (%)		56.5	77	0.005
Diabetes (%)		17.9	24	0.35
Hypertension (%)		52.1	85	0.001
Current smoker (%)		38.8	15	0.001
Heredity (%)		30.4	34	0.79
Blood glucose (mmol/L)		7.2 ± 3.3	6.28 ± 3.5	0.002
Triglycerides (mg/dL)		115.9 ± 56.8	137.1 ± 70.6	0.06
Total cholesterol (mg/dL)		188.6 ± 63.8	199.2 ± 44.5	0.11
LDL-cholesterol (mg/dL) ^a^		121.2 ± 57	119.3 ± 40.3	0.73
HDL-cholesterol (mg/dL) ^b^		47.4 ± 14.9	52.8 ± 15.6	0.068
Medical treatment at admission				
Beta blocker agents (%)		26.9	27	0.61
ACE inhibitors (%) ^c^		11.9	25	0.06
Antiplatelet agents (%)		28.2	26	0.33
Statins (%)		34.3	48	0.08
Calcium channel blocker (%)		22.4	40	0.03
Angiotensin blocker (%)		25.5	49	0.001
Antidiabetic treatment (%)		7.5	20	0.02

Data are shown as mean ± standard deviation or (%). ^a^ LDL; Low-density lipoprotein, ^b^ HDL; High-density lipoprotein; ^c^ ACE; Angiotensin-converting-enzyme.

**Table 2:** Univariate and multivariate logistic regression analyses for the risk of MI (cutoffs = tertiles).

		Unadjusted			Adjusted	
	OR	95%CI	*p* Value	OR	95%CI	*p* Value
mir_122						
t2 vs. t1	1.04	0.46–3.32	0.93	1.68	0.49–4.85	0.41
t3 vs. t1	1.45	0.65–3.23	0.36	3.16	0.87–11.4	0.08
Tertiles −7.01 and −5.58						
*p* for trend	0.36			0.08		
mir_150						
t2 vs. t1	3.04	1.59–5.28	**0.001**	3.19	1.07–6.33	**0.001**
t3 vs. t1	6.40	4.62–8.91	**0.001**	6.67	4.10–10.6	**0.01**
Tertiles −3.09 and −1.16						
*p* for trend	0.001			0.001		
mir_16						
t2 vs. t1	2.32	1.20–3.71	**0.001**	3.09	1.34–5.45	**0.002**
t3 vs. t1	4.47	3.22–5.99	**0.001**	5.27	3.32–7.96	**0.001**
Tertiles 1.90 and 3.13						
*p* for trend	0.001			0.001		
mir_186						
t2 vs. t1	2.78	1.55–4.44	**0.001**	2.33	0.76–4.38	**0.009**
t3 vs. t1	5.01	3.63–6.80	**0.001**	4.22	2.54–6.46	**0.001**
Tertiles −5.33 and −3.55						
*p* for trend	0.001			0.001		
mir_195						
t2 vs. t1	2.59	1.36–4.25	**0.001**	3.85	1.72–7.07	**0.002**
t3 vs. t1	5.24	3.81–7.08	**0.001**	5.9	3.74–9.13	**0.001**
Tertiles −4.21 and −2.40						
*p* for trend	0.001			0.001		
mir_223						
t2 vs. t1	2.21	1.08–3.60	**0.001**	1.56	0.09–3.36	**0.06**
t3 vs. t1	4.91	3.58–6.53	**0.001**	4.27	2.62–6.37	**0.001**
Tertiles −9.95 and −8.10						
*p* for trend	0.001			0.001		
mir_92a						
t2 vs. t1	4.13	2.08–8.99	**0.005**	3.66	1.21–8.72	**0.02**
t3 vs. t1	9.15	6.15–14.9	**0.001**	8.86	5.17–16.1	**0.001**
Tertiles −0.90 and 0.72						
*p* for trend	0.001			0.001		

Adjusted for gender, age, obesity, dyslipidaemia, hypertension and smoking, OR, odd ratio; CI, confidence interval.

**Table 3:** Receiver operating characteristic (ROC) curve analysis testing the contribution of each selected circulating miRNAs to the basic clinical model.

		AUC	95 % CI	χ^2^	*p* Value
miRNA					
	miR-150	0.977	0.954–0.999		
	miR-16	0.911	0.866–0.957		
	miR-186	0.922	0.877–0.967		
	miR-195	0.936	0.898–0.973		
	miR-223	0.904	0.857–0.951		
	miR-92a	0.988	0.975–1.000		
Clinical model		0.914	0.868–0.959		
+	miR-150	0.996	0.990–1.000	12.9	0.001
+	miR-16	0.981	0.965–0.997	10.3	0.0013
+	miR-186	0.973	0.953–0.994	8.5	0.0036
+	miR-195	0.986	0.973–0.998	11.3	0.001
+	miR-223	0.968	0.945–0.991	8.01	0.0045
+	miR-92a	0.999	0.998–1.000	14.1	0.001

Clinical model: gender, age, obesity, smoking, dyslipidemia and hypertension. AUC, area under the curve; CI, confidence interval; χ^2^, Chi-squared (Chi2) test.

**Table 4:** Relative miRNA expression levels of MI patients according to vital status.

	Alive (*n* = 56; 83.6%)	Dead (*n* = 11; 16.4%)	*p* Value
miRNA			
miR-150	1.271 (0.292)	1.653 (0.380)	0.578
miR-16	1.488 (0.233)	2.241 (1.054)	0.283
**miR-186**	1.493 (0.216)	4.64 (2.549)	**0.010**
miR-195	1.908 (0.457)	3.306 (2.425)	0.345
**miR-223**	1.806 (0.272)	7.096 (3.394)	**0.001**
miR-92a	1.326 (0.154)	1.397 (0.454)	0.859

**Table 5:** Prognostic effect of miRNAs on all causes of death in MI patients assessed by Cox regression analysis.

		Unadjusted			Adjusted for LVEF	
	HR	95% CI	*p* Value	HR	95% CI	*p* Value
miRNA						
miR-150	0.92	0.58–1.46	0.71			
miR-16	0.82	0.50–1.34	0.43			
miR-186	1.56	1.06–2.29	**0.025**	1.41	0.98–2.04	0.065
miR-195	0.84	0.55–1.30	0.44			
miR-223	1.75	1.19–2.57	**0.0045**	1.57	1.07–2.29	**0.02**
miR-92a	0.99	0.57–1.75	0.99			

HR; hazard ratio, CI; confidence interval, LVEF; left ventricular ejection fraction.

## Data Availability

The data presented in this study are available on request from the corresponding author.
